# Correction: Nie et al. Endoplasmic Reticulum Stress Mediated NLRP3 Inflammasome Activation and Pyroptosis in THP-1 Macrophages Infected with *Bacillus Calmette-Guérin*. *Int. J. Mol. Sci.* 2023, *24*, 11692

**DOI:** 10.3390/ijms242115824

**Published:** 2023-10-31

**Authors:** Xueyi Nie, Boli Ma, Lei Liu, Xiaotan Yuan, Mengyuan Li, Yueyang Liu, Yuxin Hou, Yi Yang, Jinrui Xu, Yujiong Wang

**Affiliations:** 1 School of Life Sciences, Ningxia University, Yinchuan 750021, China; 18794898774@163.com (X.N.); 15695013087@163.com (B.M.); liulei7@vip.163.com (L.L.); 18393712338@163.com (X.Y.); 20180044@nxmu.edu.cn (M.L.); 18395273708@163.com (Y.L.); 18209689779@163.com (Y.H.); yangyi@nxu.edu.cn (Y.Y.); 2 Key Laboratory of Ministry of Education for Conservation and Utilization of Special Biological Resources in the Western, Ningxia University, Yinchuan 750021, China

Lei Liu was not included as an author in the original publication [1].

Lei Liu’s earlier exploratory work, such as THP-1 cell culture and treatment conditions, greatly shortened our experiment time. For this reason, after a discussion with all authors, it was decided to add Lei Liu’s name after Boli Ma in this article. Initially, Lei Liu was not included in the author list, mainly because she had graduated for two years and we did not consider what the article meant to her.

The corrected Author Contributions statement appears here. The authors state that the scientific conclusions are unaffected. This correction was approved by the Academic Editor. The original publication has also been updated.
